# Supplementary material for: HAM-ART: An optimised culture-free Hi-C metagenomics pipeline for tracking antimicrobial resistance genes in complex microbial communities
Source: PLoS Genet. 2022 Mar 14;18(3):e1009776. doi: 10.1371/journal.pgen.1009776 (PMC8947609; doi:10.1371/journal.pgen.1009776)
Supplement: S1 Table — The conventional or high-antimicrobial use farms are labelled CV_1 to 5 and the organic, or low antimicrobial use farms are labelled OG_1 to 5. (PDF) [file pgen.1009776.s001.pdf]

Supplementary Table S1. Characteristics of the farms used in the study. The conventional or high-antibiotic use farms are labelled CV_1 to 5 and the organic, or low antibiotic use farms are labelled OG_1 to 5

**Farm ID**	**Location**	**Farm type**	**Total pigs**	**Date of sampling**
CV_1	Suffolk	Finishing farm	1300	02/02/2017
CV_2	Norfolk	Finishing farm	1000	09/02/2017
CV_3	Norfolk	Finishing farm	1700	16/02/2017
CV_4	Suffolk	Finishing farm	1990	16/02/2017
CV_5	Suffolk	Farrow to finish	1000	17/03/2017
OG_1	Buckinghamshire	Farrow to finish	564	26/01/2017
OG_2	Sussex	Farrow to finish	808	21/02/2017
OG_3	Hampshire	Farrow to finish	604	21/02/2017
OG_4	Wiltshire	Farrow to finish	1266	27/02/2017
OG_5	Gloucestershire	Farrow to finish	700	27/02/2017
