# Supplementary figures and images for: HAM-ART: An optimised culture-free Hi-C metagenomics pipeline for tracking antimicrobial resistance genes in complex microbial communities
Source: PLoS Genet. 2022 Mar 14;18(3):e1009776. doi: 10.1371/journal.pgen.1009776 (PMC8947609; doi:10.1371/journal.pgen.1009776)

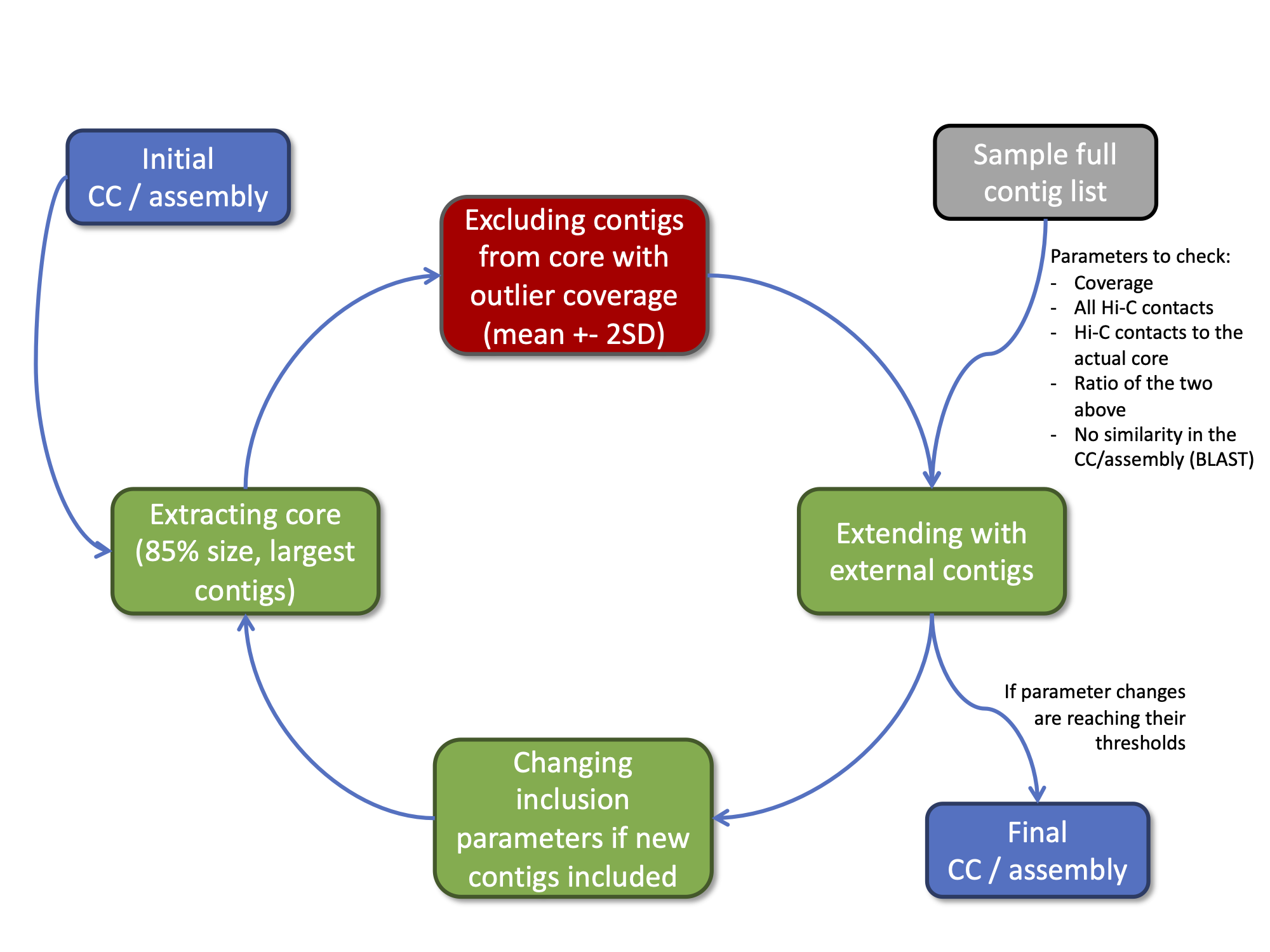

Supplement: S2 Fig — Parameter thresholds are continuously adjusted based on the contig composition of the CC / Assembly. (TIFF) [file pgen.1009776.s006.tiff]

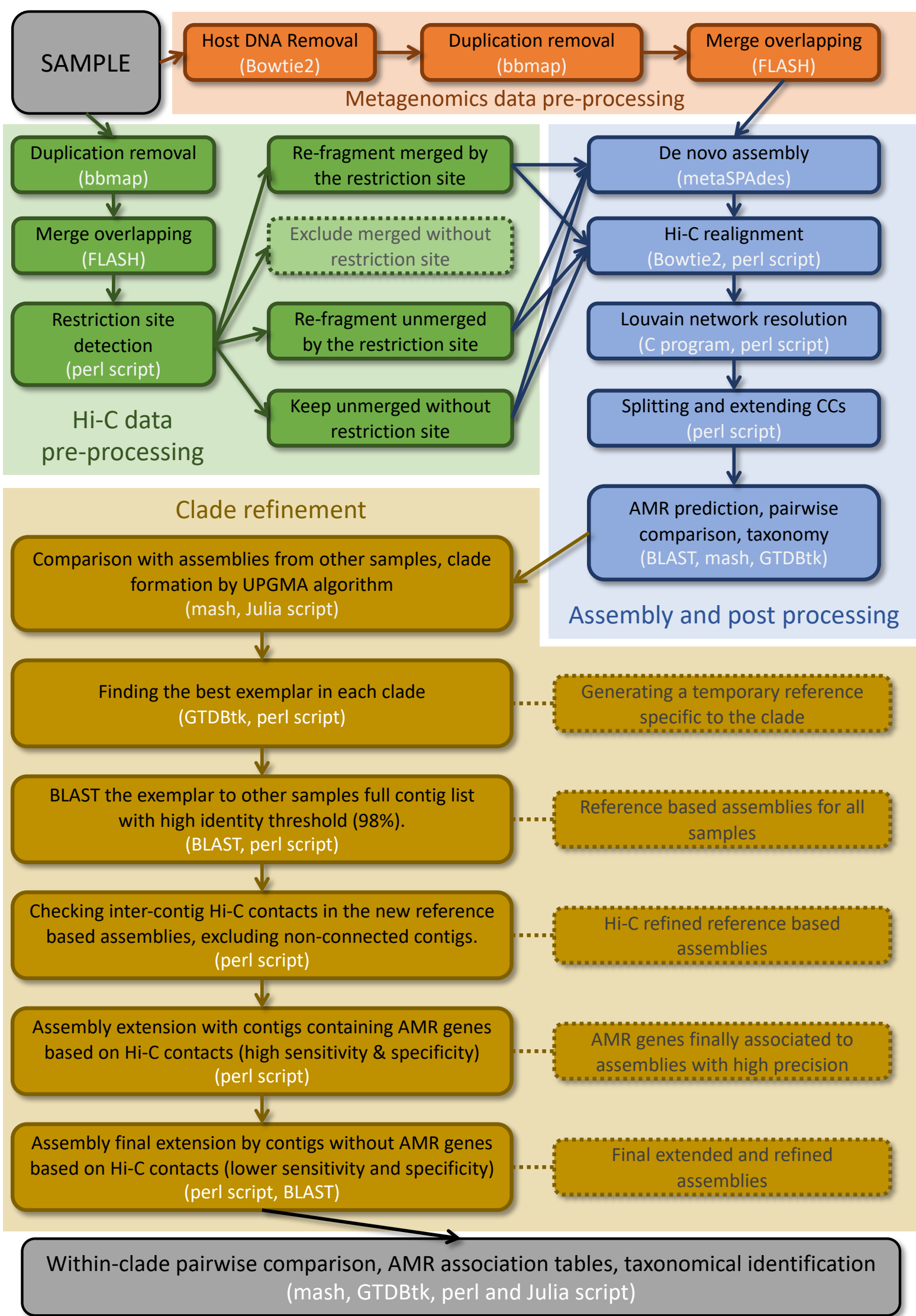

Supplement: S3 Fig — Text is coloured black for descriptions and white for the used software / script background. (PDF) [file pgen.1009776.s007.pdf]
