# Supplementary material for: HAM-ART: An optimised culture-free Hi-C metagenomics pipeline for tracking antimicrobial resistance genes in complex microbial communities
Source: PLoS Genet. 2022 Mar 14;18(3):e1009776. doi: 10.1371/journal.pgen.1009776 (PMC8947609; doi:10.1371/journal.pgen.1009776)
Supplement: Attachment — Submitted filename: ham_art_responses_final.docx [file pgen.1009776.s008.docx]

Reviewer #1: Title: HAM-ART: An optimised culture-free Hi-C metagenomics pipeline for tracking antimicrobial resistance genes in complex microbial communities.

Summary:

This is a particularly well prepared and easy to read manuscript. As a consequence, I have zero minor errors to report. As the authors have discussed, their application of Hi-C to generate MAGs and link AMRs containing sequences is not novel per se. However, their approach to Hi-C MAG refinement that leverages external information sources such as GTDBtk determined single-copy genes, is innovative. At the risk of sounding pompous, I would like to commend them for their determination in pursuing the best possible outcome from their data.

That said, there is sufficient new ground being tread within the work that much more explicit methodological detail is required for each stage (from determining consensus-cluster, splitting and refining).

Further, and although I realise this is a tall-order, this reviewer would recommend that a section comprising in-silico validation (possibly against a simulated ground-truth) be added.

RESPONSE: Current methods (e.g. Sim3C, FreeHi-C) to simulate Hi-C sequencing are based on user inputted reference genome(s). As there is no dedicated standard dataset yet to benchmark the performance of a Hi-C assisted metagenomics pipeline, we believe that the usage, especially biased usage, of these tools can really affect the final performance of the pipeline. One can easily put together a community of bacterial species that are easy to separate from each other and by that provide high accuracy. For this reason, we used our “real-word” data to bin together the MAGs and associated extrachromosomal DNA and validated our findings by isolating and sequencing individual community members. While we chose *E. coli* for this validation, as we haven’t used any known reference genome data during the analysis, there is no reason to expect worse average assembly quality of any other bacterial genome.

By ‘ground truth’ we assume the reviewer means a determination of the microbiome using conventional culture or other methodology. Unfortunately we do not have the resources to undertake this work. Like the reviewer we maintain a degree of scepticism concerning our results and have tried to add confidence with orthogonal observations wherever we could.

Lastly, I did find any mention of a code repository for this pipeline, which I would regard as mandatory even at the stage of initial submission. This would have been of great assistance and access to the code would remove part of the burden of describing the method. Altogether, this leaves me with concerns for reproducibility.

RESPONSE: We apologise for any inconvenience caused. Both the raw sequencing data and the bioinformatic code are now deposited and publicly available, links and accession numbers are included in the manuscript.


Comment:

page 17 line 218: The authors may have found that a read-based approach, which targetted resistance genes, would have had greater sensitivity. This would then modify the following discussion contrasting OG/CV presence/absence. Granted, this would not enable any subsequent gene-context analysis. Note: I see this is briefly addressed at line 370.

RESPONSE: This is a good point. The section has been extended with a sentence on the ARIBA (read-based) approach we performed to validate the AMR gene presence / absence.

page 23 line 313: Although this statement is not in hot dispute for this reviewer, I would have liked there to be a wider consideration of available exogenous variables -- that is to say a more complex regression model. I see that there are some words to this effect further down (line 327) mentioning limitations.

RESPONSE: We have only a limited amount of metadata from the pig farms, that we have used in a multiple linear regression model. The results section is extended with the results and significance of the regression analysis.

page 24 line 341: Although it provides a taxonomic hint as a side-effect of its validation analysis, the authors of checkM would probably be the first to say that it has never been meant for taxonomic assignment. I would consider revising this sentence.

RESPONSE: We agree with the reviewer, we have re-written the sentence.

page 31 line 498: Did the authors consider using or did they trial the use an existing Hi-C metagenomic binning software? Modularity based clustering algorithms (such as Louvain) suffer from a resolution limit which may have impacted the authors
capacity to extract less abundant genomes.

RESPONSE: We agree with the reviewer, that the Louvain algorithm has limitations, but we chose this algorithm as we believe it is continuously the most accepted network resolution method in 3C and Hi-C metagenomics research community (see the review “Metagenome Analysis Exploiting High-Throughput Chromosome Conformation Capture (3C) Data. By M. Marbouty and R. Koszul, 2015). In the future, we believe that with the increasing amount (and potentially better quality) of available raw data, novel methods (e.g., Leiden network resolution, neural-network based methods) should be tested and implemented if they are found to perform better. In acknowledgement of the point raised by the reviewer, in the revised manuscript we have included a short section on the limitations of the Louvain method and the prospects in the discussion.

page 31 line 507: This is potentially of interest to the tool developers within Hi-C metagenomics community. As such, more detail is required to describe this method. In particular how do the authors reconcile the variation in coverage that will be seen between core and accessory fragments when splitting?

RESPONSE: We have extended the methods section with more detail regarding this splitting step and we have described how we address the potential loss of higher coverage accessory fragments.

page 31 line 512: As with my previous comment, this step requires greater detail.

RESPONSE: We have added a new supplementary figure (S2) to the manuscript that shows the detailed workflow of the iterative CC/Assembly extension step.

page 32 line 526: The detailed description found in this section, along with the brief descriptions of previous steps, highlights the novelty of the author's approach to refine MAGs. Altogether from splitting to this step, there are a number of methodological points at which things could have gone awry. As such, algorithm validation against a ground-truth would have been greatly appreciated by this reviewer.

RESPONSE: This is covered in our earlier response on “ground truth” above and comparison / benchmarking below.

Reviewer #2: The manuscript by Kalmar et al. entitled “HAM-ART: An optimized culture-free Hi-C metagenomics pipeline for tracking antimicrobial resistance genes in complex microbial communities” describes a laboratory and a computational pipeline using Hi-C data applied to metagenomic samples. Proximity ligation-based methods (3C, Hi-C) applied to environmental samples is a relatively new field with high potential notably to study mobile genetic elements in microbial communities. In the last years several methods and pipelines have been published in this field demonstrating the increasing interest in this technology. The present manuscript takes advantage of the Hi-C data to study AMR genes and their associated MAGs in a longitudinal cohort of pigs. Not surprisingly, the authors found that a higher antibiotics usage is correlated with a higher AMR genes presence. They also develop and propose a new pipeline to analyze Hi-C data ranging from metagenomic samples.
Even if the field will benefit from new pipelines, the manuscript suffers from various problems that will need to be address before publication.

Major comments:

1 - First, the authors say in the abstract that their pipeline is also a laboratory one but I cannot determine the novelty in the preparation of the libraries compare to previous protocols. Moreover, there is no data to illustrate that their Hi-C data are of good quality in terms of 3D signal.

RESPONSE: The manuscript text has been modified in several places to emphasise that while we provide a complete and comprehensive pipeline here (laboratory and bioinformatics), most of the laboratory parts were adopted by assimilating methods and approaches from previously published studies, while the bioinformatics parts were newly developed in this study.

Several tools or calculations exist to check the quality of Hi-C data (3D ratio, q3C) and it will be of great importance to know the quality of their data and to compare them to previous published datasets.

RESPONSE: We thank the reviewer for the suggestion. We have performed additional QC on our Hi-C libraries using the qc3C tool. The results for all 20 sequencing libraries are listed in an added supplementary table (S4).

Moreover, the authors say that they have implemented several quality controls in their computational pipeline (line 581) but they do not provide them for their libraries.

RESPONSE: We thank the reviewer for highlighting this omission. We have added a supplementary table (S3) to the manuscript listing all the calculated QC values for the sequencing libraries.

2- The authors describe succinctly a new computational pipeline but the description and the content will need modifications, descriptions as well as comparison with available ones. First, the authors apply an iterative partitioning procedure using the Louvain algorithm, a procedure and a software already used in previous publications and pipeline. Then they keep group of contigs that cluster together during 100 iterations of the algorithm and they call them Consensus Clusters (CCs); a term very close from the Core Communities (CCs) used in a previous publication and in an available pipeline – MetaTOR (Marbouty et al, 2017, Baudry et al. 2019).

RESPONSE: We are aware of the origin of the CC (core community) abbreviation, we used the cited method when we developed the backbone of our pipeline, and we used the CC abbreviation in its original form in the beginning. We changed the term after presenting our results and methodology in different forums and when we received stakeholder feedback regarding the usage of the “core community” term that is already used in other contexts in microbial genomics and that it can lead to confusion / misunderstanding.

Line 507: “The separation of mixed CCs is first addressed using a coverage distribution-based separation algorithm for each CC which splits the CC if the distribution of sequencing coverage was clearly multimodal.” The authors must provide the algorithm used at this step.
Line 512 :” An iterative CC extension step was built into the pipeline at this point to extend clusters based on the Hi-C inter-contig contacts and cautiously identify contigs that should be allocated to multiple CCs.” Same as previously, details are needed here

RESPONSE: We thank the reviewer for their suggestion. A detailed description of the CC splitting and an additional supplementary figure on the iterative CC/Assembly extension are provided in the revised manuscript (see the response on the same topic for reviewer #1). The algorithm is freely available in the open access source code of the pipeline.

If the authors want to publish a new pipeline as they claim it [line 305: “ HAM-ART is the first method that is designed to cope with large sample numbers, using the most common Illumina based sequencing platform and delivering results from affordable amounts of sequencing depth.”], they must provide their code to the community and benchmark their approach with already published pipeline (bin3C, MetaTOR, HicZin …) and other datasets.

RESPONSE: As we mentioned in our response to reviewer #1 the Hi-C metagenomics field lacks a proper gold standard (ground truth) for both dataset and bioinformatics pipelines. At this stage we think that different algorithmic approaches, and different ideas would contribute to the improvement of our tools in the field, as well as the access to various Hi-C metagenomics datasets. Our manuscript does not aim to compare the HAM-ART pipeline to existing tools, primarily due to its novel modules (inclusion of Hi-C libraries in *de novo* assembly, splitting and iterative extension, clade refinement) that makes it significantly different from those. Of course, we believe that benchmarks serve a very important role for new users to find the best tool, but at this point we think the field, the pipelines and the available datasets need further improvement / iterations to reach a point when these comparisons will be relevant. We have re-written parts of our manuscript to emphasise the novelty that our pipeline introduces, and we have reduced the comparative sentences / terms. We have put the software and raw data in the public domain for other researchers to reproduce our findings and test the pipeline. Unfortunately we don’t have the resources to undertake a thorough comparison of all the major software solutions.

3- The paper will be easier to understand if the authors provide a summary table with libraries constructed, assemblies generated, binning statistics … for each sample.

RESPONSE: An additional supplementary table (S3) has been added to the manuscript summarising sequencing library related information. Information on the number of assembled MAGs from different samples is presented in supplementary table S2.

Moreover, it will be of great interest for the community to have access to the different assemblies as they can reproduce some of the outputs.

RESPONSE: The generated MAGs are available upon request (as now indicated in the revised manuscript)

4- The analysis on the lnu(A) gene is interesting but lack several controls and need further analysis. Are the contigs detected as plasmids by state of the art tools like PlasFlow or PlasmidFinder ? Can the authors check the topology of the corresponding contigs by using their Hi-C data ? If the contigs are circular, they should be able to detect the 3D signal in the corresponding contact matrices.

RESPONSE: As described in the manuscript, the lnu(A) gene was found on a ~5.6kb sequence fragment, a contig that in most *de novo* assemblies (even before Hi-C binning) assembled as one continuous sequence. As the same sequence was assembled with different sequencing origins in different samples (also described in the manuscript), it clearly points to the contig’s circular nature. Furthermore, we didn’t need any state-of-the-art plasmid prediction technique to prove its plasmid nature as we have multiple high sequence identity BLAST hits to already known plasmid sequences. We have re-written the appropriate results section accordingly.

Same remark concerning the E. coli analysis and the possible plasmid.

RESPONSE: In the case of the *E. coli* assembly and plasmid, our associated plasmid sequences were all known (again we found a high identity and high coverage BLAST homology with a known plasmid). The presence of the plasmid sequence within the *E. coli* bacteria was further validated by the colony isolate sequencing from the same sample.

Minor Comments:

- Line 102 : I would replace “assembles” by “bin” as the pipeline do not assemble the genomes but regroup contigs together.

RESPONSE: We thank the reviewer for their suggestion, we have modified the sentence accordingly.

- In general, there is some problems with the terms “assembled”, “scaffolded” and binned and it is difficult to understand, for instance for the E. coli example, the limits of the method. Is it a problem of assembly (shotgun sequencing) ? a problem of binning (not enough Hi-C reads) ?

RESPONSE: We agree with the reviewer, our pipeline doesn’t aim to do traditional scaffolding (linking contigs together in the proper genomic order), so we have replaced the term scaffold/scaffolding in the manuscript and instead we now use binning and assembly.

RESPONSE: In terms of the sensitivity limits of our method, there is already a section regarding this in the discussion. However, to address the reviewers concern, we have extended the *E. coli* results section with a sentence on the potential reasons behind the threshold.

- MLST is not defined.

RESPONSE: We thank the reviewer for highlighting this. It has been corrected in the revision.

- I have problem to understand exactly how the different assemblies were performed. Do the different Hi-C and Shotgun libraries have been mixed to perform them ? or only the shotgun ones ?

RESPONSE: The pre-processing and further usage of the different sequencing libraries are described in the “Bioinformatics pipeline – pre-processing and de-novo assembly” in detail, we have extended the section regarding the usage of the Hi-C library reads.

**Have all data underlying the figures and results presented in the manuscript been provided?**
Large-scale datasets should be made available via a public repository as described in the *PLOS Genetics* [data availability policy](http://journals.plos.org/plosgenetics/s/data-availability), and numerical data that underlies graphs or summary statistics should be provided in spreadsheet form as supporting information.

Reviewer #1: Yes

Reviewer #2: **No:** data are not already accessible and i would recommand to also submit the different assemblies generated. Code is also mandatory.

PLOS authors have the option to publish the peer review history of their article ([what does this mean?](https://journals.plos.org/plosgenetics/s/editorial-and-peer-review-process#loc-peer-review-history)). If published, this will include your full peer review and any attached files.


If you choose “no”, your identity will remain anonymous but your review may still be made public.


**Do you want your identity to be public for this peer review?** For information about this choice, including consent withdrawal, please see our [Privacy Policy](https://www.plos.org/privacy-policy).

Reviewer #1: **Yes:** Matthew Z. DeMaere

Reviewer #2: **Yes:** Martial Marbouty
