# Supplementary material for: HAM-ART: An optimised culture-free Hi-C metagenomics pipeline for tracking antimicrobial resistance genes in complex microbial communities
Source: PLoS Genet. 2022 Mar 14;18(3):e1009776. doi: 10.1371/journal.pgen.1009776 (PMC8947609; doi:10.1371/journal.pgen.1009776)
Supplement: Attachment — Submitted filename: ham_art_responses_Jan22.docx [file pgen.1009776.s009.docx]

Title: HAM-ART: An optimised culture-free Hi-C metagenomics pipeline for tracking antimicrobial resistance genes in complex microbial communities.

Reviewer #2: The revised manuscript by Kalmar appears better than the previous version. Codes and data are now available and would help scientists to reproduce their results. Precisions about their computational pipeline have been also added and their approaches appear promising. The manuscript still need minor modifications / precisions:

1- The authors have provided important statistics about their HiC libraries but never discussed about them. It could of great importance as their HiC libraries do not seem to encode more 3D signal compared to a classical 3C library. Is there a real gain in performing HiC that is more expensive than 3C ?

Authors’ response: We have added the following to the discussion – “It is reasonable to observe that our Hi-C libraries in this study do not appear to encode more 3D signal compared to a classical 3C library. The resource costs of performing 3C are lower and for some research questions this approach will be preferable.”

2- I would really appreciate that the authors submit their assemblies to the NCBI or other databases as it will allow others scientist to reproduce their results without this time-consuming assembly step. It is really easy to do and will help the whole community.

Authors’ response: We have made the assemblies openly available from the open access Apollo data store at the University of Cambridge (<https://doi.org/10.17863/CAM.80312>). Text to state this has been added to the manuscript.
